# The effect of rifaximin and lactulose treatments to chronic hepatic encephalopathy rats: An [^18^F]PBR146 in‐vivo neuroinflammation imaging study

**DOI:** 10.1002/brb3.3621

**Published:** 2024-07-05

**Authors:** Xiang Kong, Song Luo, Shi Yao Wu, Jun Zhang, Gui Fen Yang, Guang Ming Lu, Long Jiang Zhang

**Affiliations:** ^1^ Department of Radiology Jinling Hospital Affiliated Hospital of Medical School, Nanjing University Nanjing Jiangsu China; ^2^ Department of Nuclear Medicine Jinling Hospital Affiliated Hospital of Medical School Nanjing University Nanjing Jiangsu China

**Keywords:** hepatic encephalopathy, lactulose, neuroinflammation, positron emission tomography, rifaximin

## Abstract

**Introduction:**

Hepatic encephalopathy (HE) is a severe neuropsychiatric complication of liver diseases characterized by neuroinflammation. The efficacies of nonabsorbable rifaximin (RIF) and lactulose (LAC) have been well documented in the treatment of HE. [^18^F]PBR146 is a translocator protein (TSPO) radiotracer used for in vivo neuroinflammation imaging. This study investigated anti‐neuroinflammation effect of RIF or/and LAC in chronic HE rats by [^18^F]PBR146 micro‐PET/CT.

**Methods:**

Bile duct ligation (BDL) operation induced chronic HE models, and this study included Sham+normal saline (NS), BDL+NS, BDL+RIF, BDL+LAC, and BDL+RIF+LAC groups. Behavioral assessment was performed to analyze the motor function, and fecal samples were collected after successfully established the chronic HE model (more than 28 days post‐surgery). In addition, fecal samples collection and micro‐PET/CT scans were performed sequentially. And we also collected the blood plasma, liver, intestinal, and brain samples after sacrificing the rats for further biochemical and pathological analyses.

**Results:**

The RIF‐ and/or LAC‐treated BDL rats showed similar behavioral results with Sham+NS group, while the treatment could not reverse the biliary obstruction resulting in sustained liver injury. The RIF or/and LAC treatments can inhibit IFN‐γ and IL‐10 productions. The global brain uptake values of [^18^F]PBR146 in BDL+NS group was significantly higher than other groups (*p *< .0001). The brain regions analysis showed that the basal ganglia, hippocampus, and cingulate cortex had radiotracer uptake differences among groups (all *p *< .05), which were consistent with the brain immunohistochemistry results. Sham+NS group was mainly enriched in Christensenella, Coprobacillus, and Pseudoflavonifractor. BDL+NS group was mainly enriched in Barnesiella, Alloprevotella, Enterococcus, and Enterorhabdus. BDL+RIF+LAC group was enriched in Parabacteroides, Bacteroides, Allobaculum, Bifidobacterium, and Parasutterella.

**Conclusions:**

RIF or/and LAC had anti‐neuroinflammation in BDL‐induced chronic HE rats with gut microbiota alterations. The [^18^F]PBR146 could be used for monitoring RIF or/and LAC treatment efficacy of chronic HE rats.

## 1 INTRODUCTION

Hepatic encephalopathy (HE) is the most common and devastating complication with a wide range of neuropsychiatric symptoms caused by acute or chronic liver disease, which is prone to have poor prognosis and risk of short‐term mortality (Häussinger et al., 2022; Rose et al., 2020). Liver failure is associated with higher ammonia levels, inflammatory cells expansion and activation as well as increased proinflammatory cytokines, the systemic inflammation will further lead to neuroinflammation (Häussinger et al., 2022). Neuroinflammation might participate in the pathogenesis of HE leading to persisting neurological impaired manifestations (Jalan & Rose, 2022; Rose et al., 2020). Monitoring neuroinflammation in vivo by position emission tomography (PET) is a critical molecular imaging method to understand neuroinflammatory processes (Masdeu et al., 2022; van Camp et al., 2021). The translocator protein (TSPO) in activated microglia has been the preferred PET target for visualizing neuroinflammation (Alam et al., 2017; Masdeu et al., 2022; van Camp et al., 2021; Viviano et al., 2022). TSPO PET radioligands have been widely used in nervous system diseases such as stroke, Alzheimer's disease, and Parkinson's disease (van Camp et al., 2021; Viviano et al., 2022). The TSPO radioligands such as [^11^C]PK11195, [^18^F]DPA714, and [^18^F]PBR146 had been applied for neuroinflammation vivo imaging in acute or chronic HE rat models (Kong et al., 2016, 2021; Luo et al., 2018). [^18^F]PBR146 had similar effect of neuroinflammation imaging compared with [^18^F]DPA714 in chronic HE model (Kong et al., 2021). Currently, most of the therapies for HE target to reduce ammonia absorption in the gut, the ammonia‐lowering treatments by rifaximin (RIF, poorly absorbed antibiotic) and lactulose (LAC, nonabsorbable disaccharides) have been universally used for the effective remission of HE (Bass et al., 2010; Häussinger et al., 2022; Kerbert & Jalan, 2020; Rose et al., 2020). In this study, we performed bile duct ligation (BDL) in rats to induce chronic HE model, and treated with RIF or/and LAC treatment. Then we designed behavioral assessments, [^18^F]PBR146 micro‐PET/CT scan, gut microbiota, biochemical and pathological examinations to analyze cognitive function, neuroinflammation, and systemic inflammation in model rats.

## 2 METHODS

### 2.1 Animal model and treatments

All experimental protocols and procedures were conducted following guidelines for the care and use of laboratory animals and were approved by the Ethics Committee for Animal Experimentation of Jinling Hospital, Medical School of Nanjing University (approval number: 2022DZGKJDWLS‐0065). This study included 42 male Sprague‐Dawley rats weighing from 200 to 250 g. The animals were housed under the a 12 h light:12 h dark cycle with a controlled conditions (temperature 18–22°C, relative humidity 40–60%, and noise level < 60 dB). All rats accessed to commercial food and sterilized water freely, and were acclimated to the laboratory environment for 3 days before experiment. The chronic HE model was induced by bile duct ligation (BDL) surgery as previous researches (DeMorrow et al., 2021; Kong et al., 2016, 2021), and Sham group rats were performed by similar surgical operation without the bile duct ligation and abscission. The BDL rats in treated groups received RIF (MedChemExpress, New Jersey, USA) or/and LAC (Aladdin, Shanghai, China) treatments. RIF was once daily intragastrically administered at 50 mg/kg in 5.35 mL/kg saline from day 1 post‐surgery until sacrifice (Gómez‐Hurtado et al., 2018; Odena et al., 2012; Shin et al., 2017). And LAC was once daily intragastrically administered at 3.57 g/kg per day in 5.35 mL/kg saline from 20 days post‐surgery (Mendes et al., 2017). The rats of control group were once daily intragastrically administered with 5.35 mL/kg saline daily. The Sham + NS group included 8 rats. The BDL rats were divided into BDL + NS group (*n* = 18) and BDL + RIF group (*n* = 16) in 20 days post‐surgery; BDL + LAC group and BDL + LAC + RIF group were randomly separated from the two groups respectively according to the survival condition at 20th day following surgery. This study lasted for more than 28 days after surgery (DeMorrow et al., 2021).

### 2.2 Behavior studies

We performed behavior studies including rotarod test, beam walking test, and motor activity experiment at 2–3 days prior to the micro‐PET scans (Agusti et al., 2011; Jover et al., 2006; Kong et al., 2016, 2021). Rotarod test was to evaluate the motor coordination and tolerance by placing rats on an accelerating rotarod apparatus (speed increased from 6 to 40 RPM over 5 min), and we recorded the time of each rat fell off from the rotarod (Agusti et al., 2011; Kong et al., 2016, 2021). Beam walking test was used to evaluate the rats’ motor coordination ability by passing through a narrow beam to reach a dark box, and we recorded the time to cross the beam and the forelimb and hindlimb foot faults numbers (Jover et al., 2006; Kong et al., 2016, 2021). Motor activity experiment was used to evaluate locomotor and vertical activities of rats by putting the animals individually in an open‐field activity chamber for 15 min, and recorded the crossovers through the line dividing the chamber into two compartments and the rearings numbers (Kong et al., 2016, 2021). The interval time between trials was at least 5 min.

### 2.3 Radiosynthesis of ligands, micro‐PET/CT scans, and image processing

This study implemented micro‐PET/CT scans on Inveon small animal micro‐PET/CT scanner (Siemens Preclinical Solution) after behavior studies. The animals were individually fixed in the prone position after anaesthetized by isoflurane inhalation (induction: 3% and thereafter 2−2.5%) in oxygen. The radioactive tracer was [^18^F]PBR146 (N,N‐diethyl‐2‐(2‐(4‐(3‐[^18^F]fluoropropoxy)phenyl)−5,7‐dimethylpyrazolo[1,5‐a]pyrimidin‐3‐yl)acetamide) which was synthesized as previously described (Fookes et al., 2008; Kong et al., 2021). The detailed synthetic route of [^18^F]PBR146 is provided in the Supplementary Materials and Figure S1. The radiotracer was injected intravenously into the lateral tail vein. PET data were acquired firstly followed by CT data acquisition. PET acquisitions were scanned for 10 min at 50 min after radiotracer injection, and sequentially underwent CT scan for about 6–10 min to allow for coregistration of radiotracer uptake with tissues. The parameters of PET settings were as follows: slice thickness = 0.78 mm, matrix size = 128 × 128, field of view = 4 × 4 cm^2^, energy levels of acquisition: 350–650 keV (default) (Kong et al., 2021). For CT scan, the tube current and voltage were set at 500 A and 50 kV, and exposure time was 500 ms (Cochran et al., 2017; Kong et al., 2021).

Image reconstruction was performed by Inveon Research Workplace (IRW 3.0, Siemens). The PET and CT images were coregistered for correct alignment in three dimensions. We draw the global brain and some important organs (lung, heart, liver, and kidney) on the images for regions of interest quantification. The quantified radioactivity uptake of ROI was presented as percent injected dose per gram (%ID/g), obtained by dividing tissue radioactivity with injected dose assuming the tissue density is 1 g/mL. In addition, we also evaluated the regional brain area radiotracer uptake by the PMOD software (version 3.7, PMOD Technologies LTD, Zurich, Switzerland). The PET images were the manually fused with the T2‐MRI template after coregistered with CT images. The software drew 58 ROIs of brain on the PET images with reference to the MR imaging‐based atlas, and gave the corresponding radioactivity values. This processing could avoid the effect of peripheral vessels and tissues, which showed higher tracer distribution (Cochran et al., 2017; Kong et al., 2021).

### 2.4 Gut microbiota analysis

Fresh fecal samples of rats in each group were collected the day before micro‐PET/CT scans avoiding the diarrhea or loose stool. The samples were immediately frozen at −20°C and transported to laboratory within dry ice package. According to previous studies, we utilized the V3–V4 hypervariable region of 16S rRNA for polymerase chain reaction amplification to interrogate and characterize gut microbiome composition (Bajaj et al., 2019; Norman et al., 2014). The community analysis, alpha diversity and beta diversity analysis were performed for microbiota bioinformatics analysis. The details are supported in Supplementary Materials.

### 2.5 Biochemical, histopathological, and immunohistochemical characterizations

We collected the canthus blood (1–2 mL) into procoagulant tubes (GD050SG, Gongdong Medical, Zhejiang, China) in the morning of previous day before micro‐PET/CT scans for venous blood ammonia measurements immediately within 0.5–1 h. At the day after micro‐PET/CT scans which was the end of experiment, we collected blood samples (5–10 mL) of each rat from heart after anesthesia for pain relief by 10% chloral hydrate solution injected intraperitoneally with 0.4 mL/100 g of body weight. The serum samples were isolated from blood stored at −80°C to conduct the determinations of liver function and renal function indicators including total bilirubin, direct bilirubin, indirect bilirubin, total protein, albumin, globulin, alanine aminotransferase (ALT), aspartate aminotransferase (AST), alkaline phosphatase, total cholesterol, high‐density lipoprotein cholesterol (HDL‐C), low‐density lipoprotein cholesterol (LDL‐C), urea, creatinine, and uric acid by the automatic analyzer (7600, Hitachi, Japan). We further analyzed the 5‐hydroxytryptamine (5‐HT), interferon‐γ (IFN‐γ), interleukin 1β (IL‐1β), IL‐6, IL‐10, and tumor necrosis factor alpha (TNF‐α) of plasma samples by enzyme‐linked immune sorbent assay (ELISA) kits according to instructions. The same animals were transcardially perfused with saline after blood samples collected, further the liver, brain, jejunum, ileum, and colon specimens were taken out and fixed in 10% buffered formaldehyde, paraffin‐embedded, and sliced. Twenty‐four specimens of those animals with [^18^F]PBR146 micro‐PET/CT imaging were conducted hematoxylin‐eosin (H&E) staining as previous studies described (Kong et al., 2016, 2021). In addition, we performed the staining of astrocyte and microglia (brain section thickness: 4–6 μm) by immunohistochemistry of glial fibrillary acidic protein (GFAP, 1:800) and ionized calcium bindingadaptor molecule‐1 (IBA‐1, 1:500) respectively (Israel et al., 2016; Kong et al., 2021). All the histopathological and immunohistochemical slices were scaned by pathological section scanner. Morphological analysis and cell counts were performed by CaseViewer 1.4 and ImageJ software.

### 2.6 Statistical analysis

The quantitative data were expressed as mean ± standard deviation (SD). The significance among multiple groups was evaluated by one way analysis of variance (ANOVA) followed by a Newman–Keuls post hoc test. The data analysis was performed in the SPSS 16.0 statistical software (SPSS Inc, Chicago, Ill). *p* values < .05 were deemed to be statistically significant (Kong et al., 2016, 2021). The microbiota bioinformatics analyses includes rarefaction analysis, heat maps, Kruskal–Wallis test, linear discriminant analysis (LDA) effect size (LEfSe), principal coordinates (PCoA) analysis, and Shannon index of alpha diversity by vegan R package (V 2.15.3) and Quantitative Insights Into Microbial Ecology (V 1.9.1) (the details are supported in Supplementary Materials) (Li et al., 2020; Mahnert et al., 2019).

## 3 RESULTS

### 3.1 Weight, behavior studies, and biochemical results

**FIGURE 1:**
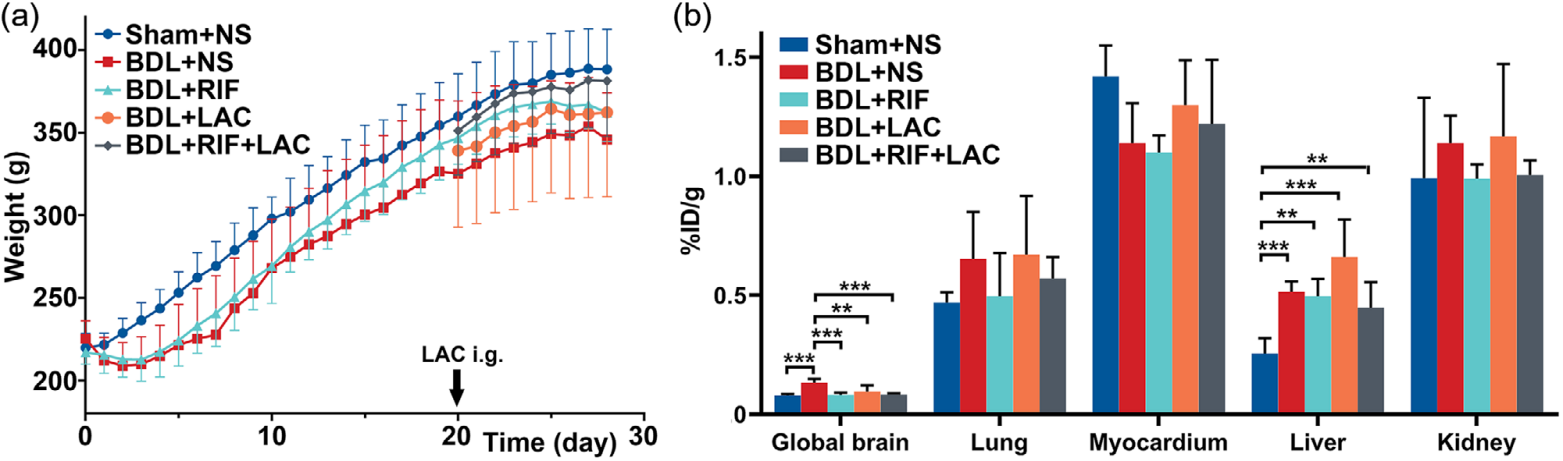
The weight changes curves (a) and the histograms of uptake values in global brain and several organs (b). The weights of Sham‐operated rats were higher than BDL‐operated rats during the study. The radiotracer uptake values of global brain of BDL+NS group was significantly higher than other groups. In addition, the liver uptake values in Sham‐operated rats were significantly lower than BDL‐operated rats, meaning the livers of BDL rats had sustained liver injury. BDL = bile duct ligation; NS = normal saline; RIF = rifaximin; LAC = lactulose; i.g. = intragastric administration. **p < .01, and ***p < .001.

All rats of Sham+NS group were survived. The mortality of BDL‐operated rats was 44.1% (15/34), mainly due to severe abdominal infection, liver failure, or gavage misoperation (the details of mortality with each group were supported in Supplementary Materials). Finally, five groups were included as follows: Sham+NS group (*n* = 8), BDL+NS group (*n* = 5), BDL+RIF group (*n* = 5), BDL+LAC group (*n* = 5), and BDL+RIF+LAC group (*n* = 4). The weight of Sham+NS group rats was higher than that of other groups (Figure 1a). The behavior results showed BDL+NS group had impairment of motor activity and tolerance compared with Sham‐operated rats, and the RIF‐ and/or LAC‐treated BDL rats showed similar behavior results with Sham+NS group (Table 1). The liver function indicators and serum ammonia levels showed significant differences among five groups (all *p *< .01, Table 1), and the RIF‐ and/or LAC‐treated BDL rats had persisted chronic liver injury. In addition, the plasma 5‐HT, IFN‐γ, and IL‐10 levels were significantly different among groups (all *p *< .05), while IL‐1β, IL‐6, and TNF‐α levels had no differences among five groups (all *p *> .05, Table 1). The 5‐HT, IFN‐γ, and IL‐10 levels in BDL+NS group were higher than those of other groups indicating that the RIF or/and LAC treatment might inhibit the inflammatory factors.

**TABLE 1:** Comparison of behavior studies, biochemical, and histopathological measurements among different groups.

Parameters	Sham+NS	BDL+NS	BDL+RIF	BDL+LAC	BDL+RIF+LAC	*p*
**Time on the rotarod (s)**	94.1 ± 22.6	72.2 ± 18.4	114.8 ± 7.9^bb^	101.8 ± 23.0^b^	114.3 ± 19.4^bb^	.022*
**Beam walking**						
Cross time (s)	15.6 ± 3.5	21.4 ± 10.5	16.2 ± 2.9	16.0 ± 4.7	13.2 ± 2.2	.190
Fault numbers	3.1 ± 1.9	3.4 ± 3.2	2.8 ± 1.9	2.6 ± 1.7	2.0 ± 1.4	.813
**Motor activity**						
Crossovers	16.5 ± 5.1^bbb^	5.2 ± 3.5^aaa^	18 ± 2.9^bbb^	10.8 ± 5.0^a b^	12.8 ± 2.1^b^	.001**
Rearings	40.5 ± 4.6^bbb^	13.2 ± 6.6^aaa^	38.3 ± 6.9^bbb^	33.6 ± 10.9^bb^	36.5 ± 12.0^bbb^	<.001***
**Biochemical measurements**						
Serum ammonia (μmol/L)	40.25 ± 11.92^bbb^	66.16 ± 7.54^aaa^	65.20 ± 8.16^aaa^	68.50 ± 8.82^aaa^	58.88 ± 9.53^aa^	<.001***
Total bilirubin (μmol/L)	0.83 ± 0.81^b^	105.48 ± 40.85^a^	143.04 ± 19.31^aaa^	102.26 ± 48.10	111.30 ± 63.53	<.001***
Direct bilirubin (μmol/L)	0.11 ± 0.10	75.68 ± 38.62	92.44 ± 30.21^a^	79.86 ± 42.94	81.97 ± 52.24	.001**
Indirect bilirubin (μmol/L)	0.72 ± 0.69^b^	29.80 ± 11.30^a^	50.60 ± 21.34^a^	22.40 ± 6.73^a^	29.33 ± 20.82	<.001***
Total protein (g/L)	61.96 ± 3.58^bbb^	73.80 ± 5.32^aaa^	61.52 ± 5.00^bb^	68.34 ± 6.17^a^	67.03 ± 1.60	.002**
Albumin (g/L)	30.39 ± 1.48^b^	27.20 ± 1.42^a^	22.32 ± 2.79^a^	23.54 ± 4.48	27.70 ± 4.20	.001**
Globulin (g/L)	31.57 ± 2.57^bbb^	46.60 ± 4.45^aaa^	39.20 ± 3.63^aa bb^	44.80 ± 3.11^aaa^	39.33 ± 5.69^aa b^	<.001***
ALT (U/L)	40.71 ± 10.81^bb^	88.00 ± 26.06^aa^	62.60 ± 19.97	84.80 ± 24.30^aa^	62.67 ± 14.84	.003**
AST (U/L)	110.43 ± 44.21	602.40 ± 246.76	475.80 ± 83.93^aa^	449.00 ± 119.81^a^	390.00 ± 224.89	<.001***
Alkaline phosphatase (U/L)	2.29 ± 1.38	13.20 ± 10.62	15.60 ± 5.32^a^	12.00 ± 3.54^a^	18.33 ± 10.21	.006**
Total cholesterol (μmol/L)	1.89 ± 0.20^bb^	3.25 ± 0.37^aa^	3.26 ± 0.96	3.37 ± 1.11	2.81 ± 0.17^aa^	.006**
HDL‐C (μmol/L)	0.80 ± 0.09^b^	0.61 ± 0.20^a^	0.45 ± 0.09^aa^	0.51 ± 0.17^aa^	0.52 ± 0.23^a^	.006**
LDL‐C (μmol/L)	0.22 ± 0.06	1.37 ± 0.58	1.22 ± 0.27^aa^	1.20 ± 0.29^aa^	1.07 ± 0.36	<.001***
Urea (μmol/L)	6.20 ± 0.55	6.76 ± 2.02	5.08 ± 0.33	6.30 ± 1.52	5.23 ± 0.38	.187
Creatinine (μmol/L)	29.76 ± 5.89	25.54 ± 4.10	20.68 ± 4.73^aa^	25.82 ± 1.49	20.10 ± 1.64^aa^	.011*
Uric acid (μmol/L)	42.71 ± 31.85^bb^	85.00 ± 24.38^aa^	73.60 ± 13.78^a^	72.20 ± 24.53	47.67 ± 14.01^b^	.048*
5‐HT (pg/mL)	287.91 ± 23.01^bb^	381.51 ± 36.98^aa^	347.85 ± 59.95	345.49 ± 58.41	372.34 ± 60.43^a^	.039*
IFN‐γ (pg/mL)	1218.73 ± 350.04^bb^	1717.03 ± 199.66^aa^	1562.69 ± 153.94^a^	1661.11 ± 151.37^aa^	1519.59 ± 282.71	.024*
IL‐1β (pg/mL)	27.60 ± 6.22	35.01 ± 4.11	34.34 ± 5.02	32.77 ± 5.01	31.70 ± 3.50	.147
IL‐6 (pg/mL)	70.67 ± 15.53	107.12 ± 10.17	87.39 ± 26.10	89.63 ± 24.13	82.33 ± 8.59	.059
IL‐10 (pg/mL)	67.63 ± 10.55^bbb^	96.06 ± 6.56^aaa^	82.06 ± 15.37^a^	81.62 ± 14.58	82.82 ± 2.87	.011*
TNF‐α (pg/mL)	248.74 ± 74.35	301.13 ± 64.66	265.42 ± 56.81	274.80 ± 60.60	264.54 ± 53.42	.743
**No. of microglia (cells/mm^2^)**†	26.93 ± 5.08	35.07 ± 3.19	31.40 ± 3.34	32.00 ± 2.62	29.95 ± 4.19	.193

*Note*: Values are the mean ± standard deviation from at least three rats each group. Values significantly different from Sham+NS group are indicated by “a” and from BDL+NS group by “b.” *p < .05, **p < .01, and ***p < .001; ^a^ *p *< 0.05, ^aa^ *p *< .01, and ^aaa^ *p *< .001; ^b^ *p *< .05, ^bb^ *p *< .01, and ^bbb^ *p *< .001 were regarded as statistically significant.

BDL = bile duct ligation; NS = normal saline; RIF = Rifaximin; LAC = Lactulose; ALT = alanine aminotransferase; AST = aspartate aminotransferase; HDL‐C = high‐density lipoprotein cholesterol; LDL‐C = low‐density lipoprotein cholesterol; 5‐HT = 5‐hydroxytryptamine; IFN‐γ = interferon‐γ; IL‐1β = interleukin 1β; IL‐6 = interleukin 6; IL‐10 = interleukin 10; TNF‐α = tumor necrosis factor alpha.

†IBA‐1 positive microglia cells were counted in the basal ganglia.

### 3.2 Micro‐PET/CT results

A total of 24 rats (*n* = 4 for BDL+RIF+LAC group, *n* = 5 for other 4 groups) were involved in micro‐PET/CT imaging on two consecutive days because of the short half‐life of [^18^F]PBR146. The injected radioactivity of [^18^F]PBR146 was not significantly different among groups [*p *= .380, Sham+NS (253.5 ± 23.4 μci, BDL+NS (287.1 ± 12.3 μci), BDL+RIF (281.2 ± 51.2 μci), BDL+LAC (274.6 ± 36.1 μci), and BDL+RIF+LAC (253.4 ± 21.3 μci)].

**FIGURE 2:**
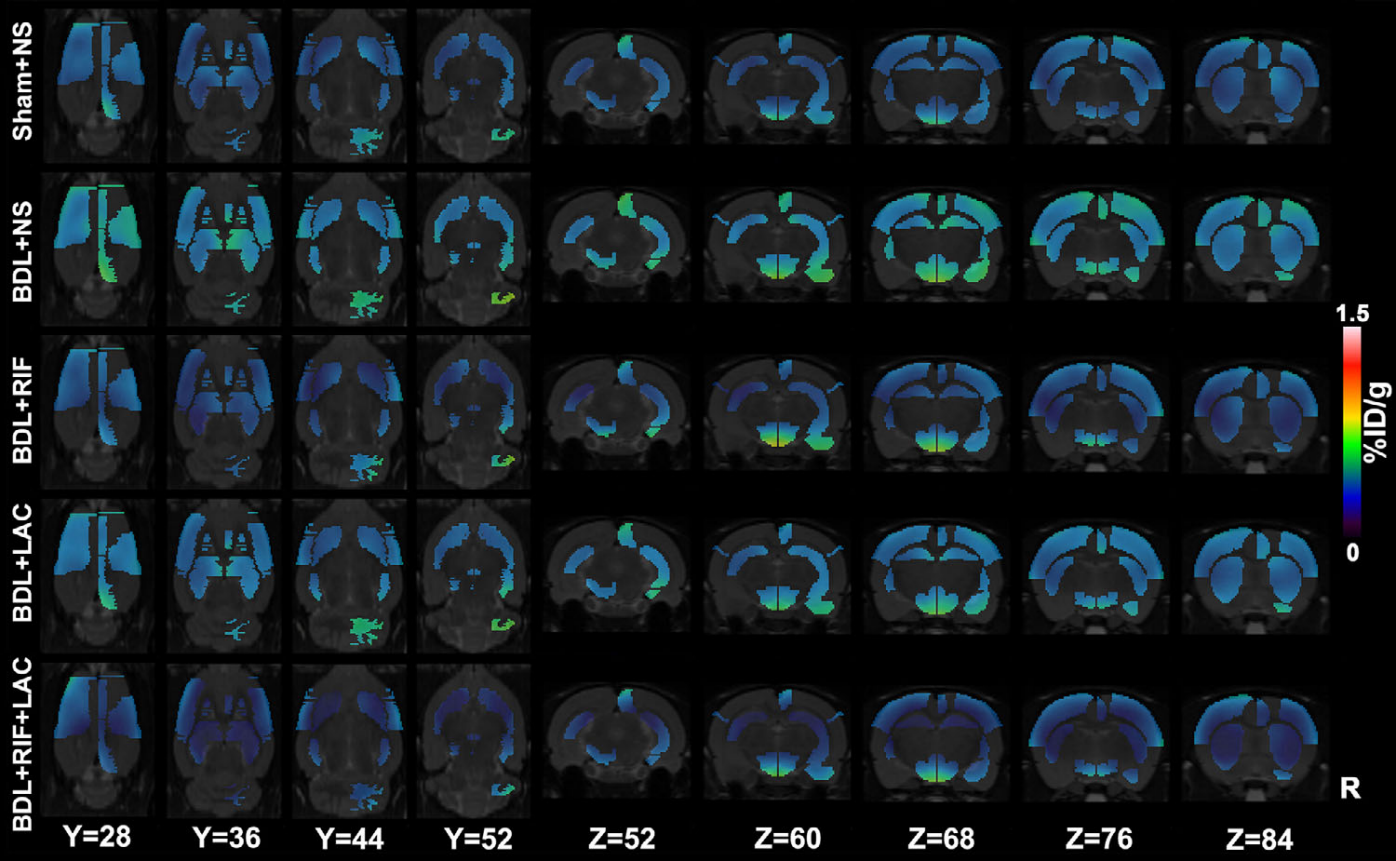
Representative [^18^F]PBR146 micro‐PET/CT images in five groups. The significant differences of regional brain regions were located in bilateral accumbens, striatum, anterodorsal hippocampus, hypothalamus, right amygdala, cingulate cortex, medial prefrontal cortex, retrosplenial cortex, posterior hippocampus, left motor cortex, somatosensory cortex among five groups. The uptake values of these regions in RIF and/or LAC‐treated BDL rats were similar as Sham+NS group, while revealed lower %ID/g than NS‐treated BDL rats. BDL = bile duct ligation; NS = normal saline; RIF = rifaximin; LAC = lactulose; R = right.

The global brain average %ID/g value of [^18^F]PBR146 in BDL+NS group was significantly higher than other groups (Figure 1b, Table S1). There were no difference of [^18^F]PBR146 uptake values in lung, myocardium, and kidney among groups (all *p *> .05), while the uptake values of liver in Sham+NS group were significantly lower than other groups (*p *< .001, Figure 1b, Table S1). Several regions showed significant differences among groups mainly including bilateral accumbens, striatum, anterodorsal hippocampus, hypothalamus, right amygdala, cingulate cortex, medial prefrontal cortex, retrosplenial cortex, posterior hippocampus, left motor cortex, and somatosensory cortex (all *p *< .05, Figure 2, Table S1). The uptake values of these regions in RIF‐ and/or LAC‐treated BDL rats were lower than those of BDL+NS groups, which were similar as those of Sham+NS group.

### 3.3 Histopathological, and immunohistochemical characterizations

**FIGURE 3:**
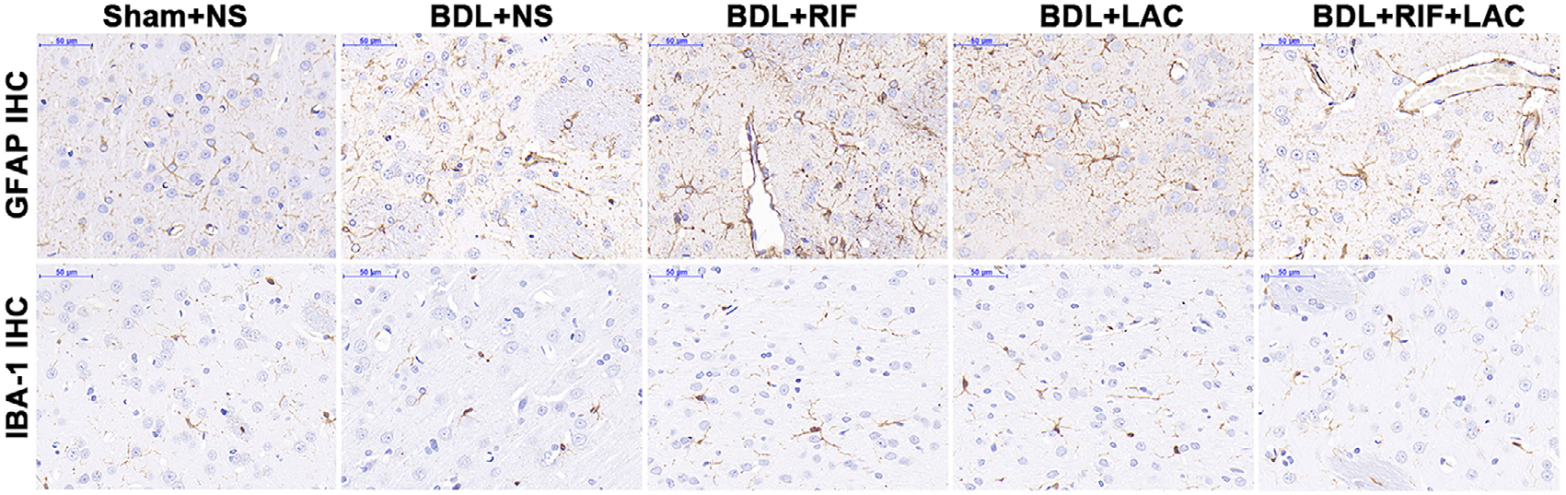
Representative IHC findings of different groups. The IBA‐1 IHC micrographs of microglia of Sham rats as well as RIF‐ and/or LAC‐treated BDL rats showed ramified shapes (resting microglia), while NS‐treated BDL rats showed amoeboid shapes (activated microglia) in basal ganglia. IHC = immunohistochemistry; BDL = bile duct ligation; GFAP = glial fibrillary acidic protein; IBA‐1 = ionized calcium‐binding adapter molecule 1; NS = normal saline; RIF = rifaximin; LAC = lactulose.

The liver weight of Sham+NS group (16.80 ± 1.14 g) was significantly lighter than other groups [BDL+NS (26.75 ± 4.95 g), BDL+RIF (28.71 ± 4.55 g), BDL+LAC (28.19 ± 9.42 g), and BDL+RIF+LAC (24.84 ± 8.55 g); *p *= .007]. The hepatic and H&E staining findings showed chronic liver impairments with expanded bile duct leading and destroyed hepatic cords with inflammatory infiltration in BDL‐operated rats, while the brain and intestinal H&E staining results had no differences between Sham and BDL rats (Figure S2). The morphological analysis of microglia with IBA‐1 immunohistochemistry showed that microglia of RIF‐ and/or LAC‐treated BDL rats as well as Sham+NS group were ramified, while the BDL+NS rats showed ameboid shape (Figure 3), which were consistent with the [^18^F]PBR146 micro‐PET/CT findings. Although the amounts of immune reactive microglia in basal ganglia had no significant difference among five groups (*p *= .193, Table 1), the RIF‐ and/or LAC‐treated BDL rats had slightly lower amounts of microglia than NS‐treated BDL rats.

### 3.4 Analysis of gut microbiota

**FIGURE 4:**
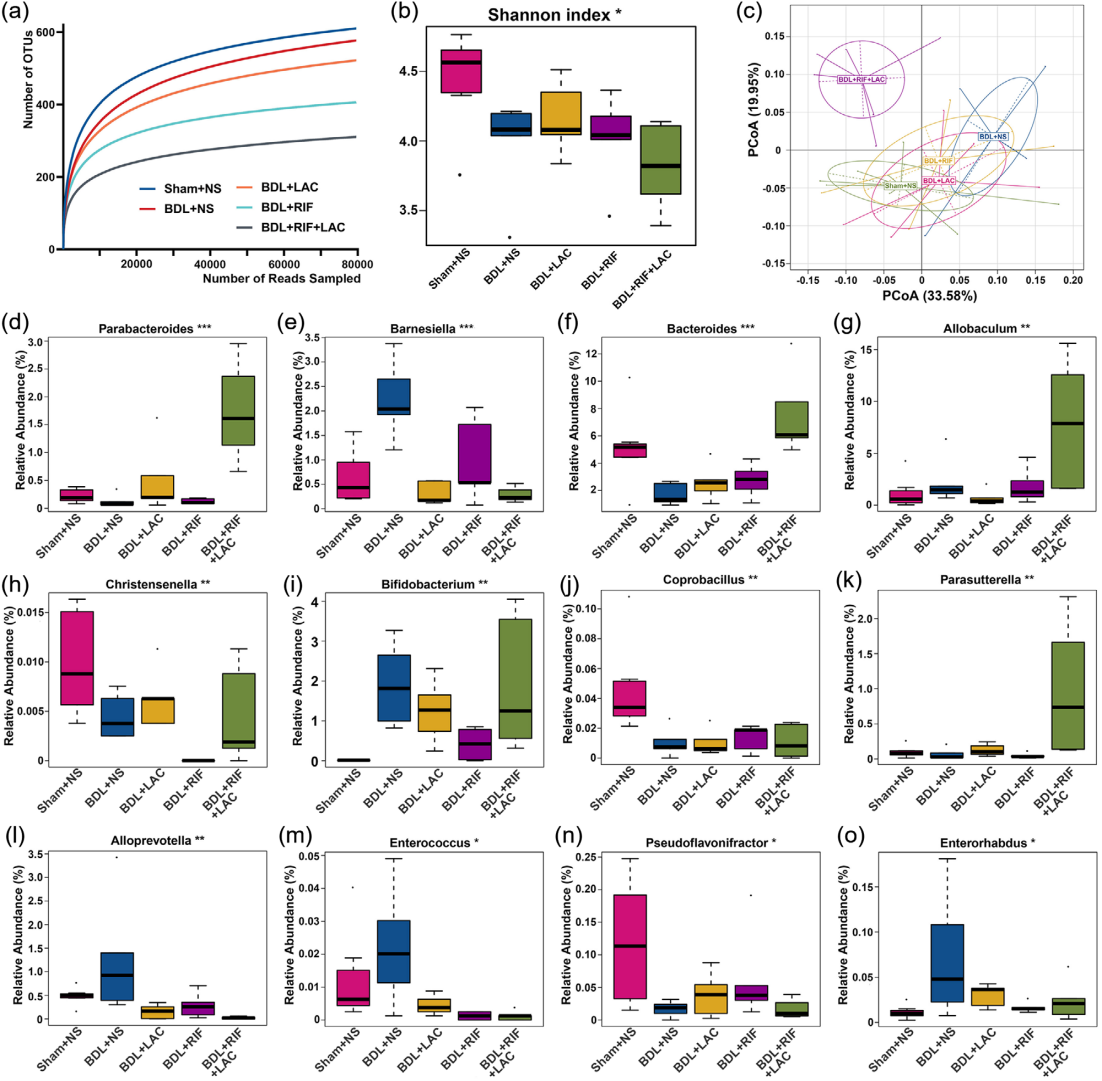
The gut microbiota analysis results. Rarefaction curves for OTUs of five groups (a). Comparison of microbial alpha diversity by the Shannon index based on genus profiles among five groups (b). Principal coordinate analysis of samples of five groups (c). Relative abundances of several genera that showed significant differences among five groups (d–o). In box plots (b and d–o), boxes represent the IQRs between the first and third quartiles, and the line inside the box represents the median; whiskers represent the lowest and highest values within 1.5 times IQR from the first and third quartiles; and the dots outside whiskers represent the outliers. OTUs = operational taxonomic units; BDL = bile duct ligation; NS = normal saline; RIF = rifaximin; PCoA = principal coordinate analysis; IQRs = interquartile ranges. *p < .05, **p < .01, and ***p < .001 were regarded as statistically significant.

The top 100 relative abundances community compositions of samples among five group were similar (Figure S3A). The rarefaction analysis showed the sample reads richness was the highest in Sham+NS group and the lowest in BDL+RIF+LAC group (Figure 4a). Shannon index of alpha diversity showed significant difference among five groups (*p *= .024, Kruskal–Wallis rank‐sum test, Figure 4b); the diversity of Sham+NS group was higher than that of other groups. The beta diversity with LDA in LefSe differential analysis showed that the communities with LDA score higher than 2 in Sham+NS group were richer than other groups based on genus profiles (Figure S3B). Ordination analysis of weighted unifarc PCoA based on the operational taxonomic units (OTUs) revealed separation of BDL+RIF+LAC group and other groups and the separation between Sham+NS group and BDL+NS group (Figure 4c), while the ANOSIM results revealed a more heterogeneous community structure in BDL+RIF+LAC group than Sham+NS group (*R* = 0.655, *p* = .0001, Figure S3C). The ANOVA analysis based on genus profiles showed that Sham+NS group was mainly enriched in Christensenella, Coprobacillus, and Pseudoflavonifractor; BDL+NS group was mainly enriched in Barnesiella, Alloprevotella, Enterococcus, and Enterorhabdus; and BDL+RIF+LAC group was enriched in Parabacteroides, Bacteroides, Allobaculum, Bifidobacterium, and Parasutterella (Figure 4d–o).

## 4 DISSCUSSION

[^18^F]PBR146 has been proven to in vivo monitor the neuroinflammation alterations for chronic HE in BDL rats (Kong et al., 2021). The widely empirical pharmacological approaches to HE consist of RIF and LAC, which have formed the mainstay of guidelines for management of HE (Acharya & Bajaj, 2018; Nardelli et al., 2023; Riordan & Williams, 2010). The nonabsorbable for intestine of RIF and LAC could lead to gut microbiota composition changes (Acharya & Bajaj, 2018; Gitto et al., 2023). In this study, we performed [^18^F]PBR146 micro‐PET/CT to observe the RIF or/and LAC curative efficacies to BDL rats and further investigated the gut microbiota alterations. It was found that RIF or/and LAC had anti‐neuroinflammation for BDL rats that could be imaged by [^18^F]PBR146 micro‐PET/CT accompanying with microbiota alterations of fecal samples.

Due to extensive and continuous liver injury, the BDL rats lost body weight gradually but with compensatory hypertrophy of liver, which had 44% (15/34) mortality and chronic liver pathological changes. Besides, the RIF or/and LAC treatments to BDL‐operated rats could not improve the liver function indicators and serum ammonia levels (Kong et al., 2016, 2021), while the renal function analysis suggested that RIF treatment to chronic hepatic injury could increase the creatinine clearance similar to previous study (Hanafy & Hassaneen, 2016). The behavioral assessment results showed that RIF or/and LAC treatments could improve the motor coordination, tolerance, and activities to BDL rats. We also found the plasma inflammatory factor levels of IFN‐γ and IL‐10 were reduced in RIF or/and LAC‐treated BDL rats.

The uptake values of [^18^F]PBR146 of global brain in BDL+NS group were significantly higher than other groups, especially in the bilateral basal ganglia, hypothalamus, and hippocampus regions, which were consistent with IHC changes of brain tissues. Although the amount of microglia in basal ganglia had no significant difference, the morphology of microglia showed ramified shape in RIF and/or LAC‐treated BDL rats as well as Sham+NS group. These findings were partially consistent with previous studies (Kong et al., 2016, 2021; Luo et al., 2018). Basal ganglia had been proved to be related with brain function and structure in HE (Zhang et al., 2013), and hippocampus was related with cognitive functions including learning and memory (Izquierdo‐Altarejos et al., 2023). Both RIF and LAC proved effective for improving motor ability and reducing neuroinflammation in BDL rats. And RIF concomitantly with LAC or RIF single agent had more effectively than LAC alone for BDL rats. Mendes et al. (2017) treated BDL rats with LAC once a day for 7 days, and they found that LAC could reduce hyperammonemia and neuronal hyperactivity in brain areas related to motor behavior. Rahimi et al. (2014) reported that standard LAC therapy might be inferior to polyethylene glycol 3350‐electrolyte solution treatment in hospitalized cirrhosis patients for acute HE. These studies were partly consistent with our study, while LAC treatment was not superior to RIF treatment in BDL rats, it may be due to short treatment time (8–9 days) of LAC in our study. Bass et al. (2010) observed 299 patients for 6 months and found that RIF treatment maintained remission from HE and reduced the risk of hospitalization of HE. Another study by Shin et al. (2017) reported that RIF did not reduce inflammation and fibrosis in BDL rat model. While the present study found that RIF alone or RIF+LAC treatments could reduce the plasma inflammatory indicators levels for BDL rats, this might be because the BDL models were maintained for more than 28 days and were administered with RIF daily continued to the end of the experiment which were different from Shin et al.’s (2017) research. Meng et al. (2022) found RIF could prevent cognitive deficit of circadian rhythm disruption mice through protecting the gut barrier and ameliorating neuroinflammation, which were coincide with our results.

In addition, the gut microbiota alterations were observed among groups in this study. The level of Barnesiella, which was closely related to bile acids, was higher in BDL+NS group than other groups consistent with Jiang et al.’s (2020) research. Christensenellaceae was enriched in Sham+NS group and increased relatively in BDL+RIF+LAC group; it was a kind of Firmicutes bacteria, an important player in organisms’ health (Waters & Ley, 2019). Coprobacillus and Pseudoflavonifractor decreased and Alloprevotella, Enterococcus, and Enterorhabdus increased relative abundances in BDL+NS group might be related to the dysbiosis and bacterial translocation of chronic liver failure (Li et al., 2020; Wang et al., 2020). We also found RIF‐ and LAC‐treated BDL rats reversed the gut microbiota dysbiosis by increasing the relative abundances of potentially beneficial bacteria, such as Parabacteroides, Bacteroides, Bifidobacterium, Allobaculum, and Parasutterella (Carbajo‐Pescador et al., 2019; Ju et al., 2019; Li et al., 2020; van Hul et al., 2018).

Taken together, the efficacies of RIF or/and LAC treatments for chronic HE model were mutually validated through multi‐dimensional analysis of behavior, micro‐PET/CT imaging, histopathology, and gut microbiota analysis in this study. The speculation of causality among these factors was that the gut microbiota alterations affect brain tissue histopathological and imaging abnormalities through gut–brain axis, leading to chronic HE symptoms (behavioral impairments) (Butterworth, 2013).

Several limitations should be acknowledged. First, the sample sizes of each group were relatively small because of BDL rats with higher mortality, and higher cost of micro‐PET/CT scanning forced us to reduce the numbers of scanning models. Second, the inflammatory factors and metabolism in brain tissue should be further researched. Third, the gut microbiomics should be further analyzed to investigate the relationships of microbiota metabolites and neuroinflammation. Fourth, the immunohistochemistry of GFAP and IBA‐1 showed weak; we would improve the staining technique. Fifth, the functional connectivity of brain regions with differences among groups should be taken into account in our further research.

## 5 CONCLUSIONS

The RIF and LAC treatments have inhibitory effects to neuroinflammation in chronic HE rats associated with motor functions improvement and gut microbiota alterations. The [^18^F]PBR146 micro‐PET/CT imaging is helpful to noninvasively observe the RIF and LAC treatment efficacy on neuroinflammation of BDL‐induced chronic HE.

## AUTHOR CONTRIBUTIONS

GML and LJZ conceived and designed the study. XK, SL, and SYW contributed to the literatures search. XK, JZ, SYW, and GFY completed the experiment and the writing and revision of this manuscript. All authors approved it for publication.

## CONFLICT OF INTEREST STATEMENT

All authors declare that they have no competing interests.

### PEER REVIEW

The peer review history for this article is available at https://publons.com/publon/10.1002/brb3.3621.

## Supporting information

Supporting information

## Data Availability

The data that support the findings of this study are available from the corresponding author upon reasonable request.
