# Supplementary material for: The effect of rifaximin and lactulose treatments to chronic hepatic encephalopathy rats: An [18F]PBR146 in‐vivo neuroinflammation imaging study
Source: Brain Behav. 2024 Jul 5;14(7):e3621. doi: 10.1002/brb3.3621 (PMC11226542; doi:10.1002/brb3.3621)
Supplement: Supplementary file 1 — Supporting information [file BRB3-14-e3621-s001.docx]

**Supplementary materials**

**Materials and Methods**

## Radiosynthesis of [^18^F]PBR146

The synthetic route of [^18^F]PBR146 [N,N-diethyl-2-(2-(4-(3-[^18^F]fluoropropoxy)phenyl)-5,7-dimethylpyrazolo[1,5-a]pyrimidin-3-yl)acetamide] was illustrated in **Supplementary figure S1A** referring to previous study ^[1, 2]^. **Step 1:** Methyl 4-hydroxybenzoate (9.615 g, 63.2 mmol) and anhydrous potassium carbonate (30.570 g, 221 mmol) was slowly treated with 150 mL acetone and 2-bromopropane (11.660 g, 94.8 mmol). And the mixture was sealed at 85 °C for 72 h followed by concentrated and separated by chromatography column (PE:EA=20:1), then obtained 11.627 g colourless liquid (Compound 1) with yield 94.7%. ^1^H NMR (300 MHz, CDCl_3_) δ 7.97 (2H, d, J=9.0 Hz), 6.88 (2H, d, J=8.9 Hz), 4.66-4.58 (1H, sep), 3.87 (3H, s), 1.35 (6H, d, J=6.0Hz), LC-MS: calculated for C_11_H_14_O_3_, 194.23, found [M+H] 195.1. **Step 2:** Compound 1 (11.627 g, 59.86 mmol) in methylbenzene (60 mL) was cooled to 0 °C followed by 60% sodium hydride (4.789 g, 119.72 mmol), heated to 80 °C stirred for 10 min, and then dropped with acetonitrile (12.287 g, 299.68 mmol) to methylbenzene heated to 85 °C for 16-18 h. After cooling, the solution was added with n-hexane to stir and filter, and the filtrate was dissolved in 500 mL ice water followed by extracting by ethyl acetate (250 mL*2), then successively dried, concentrated and separated by chromatography column (PE:EA=10:1) to give a white solid (Compound 2, 9.791 g, 80.5%). ^1^H NMR (300 MHz, CDCl_3_) δ 7.88 (2H, d, J=8.9 Hz), 6.93 (2H, d, J=8.9 Hz), 4.71-4.63 (1H, sep), 4.01 (2H, s), 1.38 (6H, d, J=6.1 Hz), LC-MS: calculated for C_12_H_13_NO_2_, 203.24, found [M+H] 204.1. **Step 3:** Sodium hydroxide (0.455 g, 10.94 mmol) and ethylalcohol (80%, 80 mL) were stirred for 15 min at room temperature, then the mixture was added sodiumiodide (4.441 g, 29.63 mmol), Compound 2 (2.180 g, 9.94 mmol), and N,N-diethylacetamide (1.488 g, 9.94 mmol) successively at 20-25 °C for 16-18 h followed by concentrated and separated by chromatography column (n-Hexane:EA=10:1~8:1~5:1~3:1) to afford an oily product (Compound 3, 2.467 g, 72.7%). ^1^H NMR (300 MHz, CDCl_3_) δ 8.02 (2H, d, J=9.0 Hz), 6.95 (2H, d, J=9.0 Hz), 5.00 (1H, dd, J=9.2, 4.6 Hz), 4.71-4.63 (1H, sep), 3.44-3.38 (4H, m), 2.85 (1H, dd, J=16.1, 4.6 Hz), 1.38-1.36 (6H, d, J=6.0 Hz), 1.29-1.23 (3H, t, J=7.1 Hz), 1.11-1.06 (3H, t, J=7.1Hz), LC-MS: calculated for C_18_H_24_N_2_O_3_, 316.40, found [M+H] 317.2. **Step 4:** Compound 3 (2.460 g, 7.77 mmol) and ethylalcohol (80%, 50 mL) were added with 80% hydrazine hydrate (0.973 g, 15.55 mmol) and acetic acid (0.747 g, 12.44 mmol) boiled to 100 °C with reflux reaction for 4 h, then concentrated up to dryness. After cooling, the solution was added with 50 mL 2N hydrochloric acid and 50 mL diethyl ether stirred for 20 min separated out off-white solid, then the filtrate was washed with diethyl ether and added with 100 mL water followed by basifing to pH 12 with 50 mL 1N sodium hydroxide and extracting by dichloromethane (100 mL*2) to dryness, and collected 1.888 g off-white solid (Compound 4, 73.5%). ^1^H NMR (300 MHz, CDCl_3_) δ 7.28 (2H, d, J=8.7 Hz), 6.94 (2H, d, J=8.7 Hz), 4.63-4.55 (1H, sep), 3.50 (2H, s), 3.33 (2H, q, J=7.2 Hz), 3.16 (2H, q, J=7.2 Hz), 1.36 (6H, d, J=6.1 Hz), 1.08 (3H, t, J=7.1 Hz), 0.92 (3H, t, J=7.1 Hz); LC-MS: calculated for C_18_H_26_N_4_O_2_, 330.43, found [M+H] 331.1. **Step 5:** Compound 4 (1.888 g, 5.7 mmol) and ethylalcohol (80%, 30 mL) were added with acetylacetone (0.572 g, 5.7 mmol) for reflux reaction at 90 °C for 16-18 h. After concentration, the mixture was recrystallized with isopropanol to obtain a white spumescence solid (Compound 5, 1.530 g, 67.8%). ^1^H NMR (300 MHz, CDCl_3_) δ 7.75 (2H, d, J=8.8 Hz), 6.96 (2H, d, J=8.8 Hz), 6.49 (1H, s), 4.64-4.56 (1H, sep), 3.91 (2H, s), 3.50 (2H, q, J=7.2 Hz), 3.41 (2H, q, J=7.0 Hz), 2.74 (3H, s), 2.53 (3H, s), 1.36-1.34 (6H, d, J=6.0 Hz), 1.20 (3H, t, J=7.1 Hz), 1.11 (3H, t, J=7.1 Hz), LC-MS: calculated for C_23_H_30_N_4_O_2_, 394.52, found [M+H] 395.2. **Step 6:** Compound 5 (5.268 g, 13.4 mmol) and 100 mL dichloromethane were added with anhydrous aluminum chloride (5.876 g, 44.1 mmol) in ice bath followed by reflux reaction at 30 °C for 2 h. After cooling to 0 °C, the mixture was added with saturated ammonium chloride solution and stirred for 30 min to quench, and then extracted by dichloromethane (100 mL*2) to dryness, and separated by chromatography column (DCM:MeOH=30:1) to give a white solid (Compound 6, 3.627g, 77.1%). ^1^H NMR (300 MHz, CDCl_3_) δ 7.65 (2H, d, J=8.6 Hz), 7.30 (1H, brs), 6.82 (2H, d, J=8.6 Hz), 6.49 (1H, s), 3.96 (2H, s), 3.51 (2H, q, J=7.1 Hz), 3.38(2H, q, J=7.2 Hz), 2.73 (3H, s), 2.53 (3H, s), 1.15 (3H, t, J=7.1 Hz), 1.08 (3H, t, J=7.1 Hz), LC-MS: calculated for C_20_H_24_N_4_O_2_, 352.44, found [M+H] 353.2. **Step 7:** Compound 6 (0.300 g, 0.85 mmol) in anhydrous potassium carbonate (0.413 g, 2.99 mmol) was slowly treated with acetonitrile (10 mL) followed by F(CH_2_)3OTS (0.198 g, 0.85 mmol) and the mixture sealed at 80 °C for 16-18 h. The reaction solution was poured into water extracted with ethyl acetate (80 mL*2), then the concentrate was separated by chromatography column (DCM:MeOH=100:1~50:1) followed by ether evaporated, further purified by chromatography column (dichloromethane:methanol=100:1~50:1~30:1) afford 0.342 g off-white solid. Eventually, the solid was recrystallized from ethyl acetate and n-hexane to give the PBR146 (Compound 7) as white solid (0.221 g, 63.0%). ^1^H NMR (300 MHz, CDCl_3_) δ 7.75 (2H, d, J=8.7 Hz), 6.98 (2H, d, J=8.7 Hz), 6.50 (1H, s), 4.66 (2H, dt, J=47.1, 5.8 Hz), 4.15 (2H, t, J=6.1 Hz), 3.91 (2H, s), 3.51(2H, q, J=7.2 Hz), 3.41 (2H, q, J=7.1 Hz), 2.74 (3H, s), 2.53 (3H, s), 2.21 (2H, dm, J=26.0 Hz), 1.20 (3H, t, J=7.1Hz), 1.11 (3H, t, J=7.1 Hz), LC-MS: calculated for C_23_H_29_FN_4_O_2_, 412.51, found [M+H] 413.4. In addition, the [^18^F]fluoride labeled procedure to PBR146 was similar with [^18^F]DPA-714. The final product was analyzed by HPLC quality control of [^18^F]PBR146. [^18^F]PBR146 has similar retention time of absorption peak in HPLC with [^19^F]PBR146, but the peak magnitude was different (**Supplementary figure S1B and C**). Synthetic time was 60 min, and radiochemical yield of [^18^F]PBR146 was of 63.0%. Radiochemical purity was > 95% and the specific activity ranged from 85.7 to 104.9 GBq/μmol.

## Gut microbiota analysis

The gut bacterial DNA was extracted at Genesky Biotechnologies Inc., Shanghai, using kits (TransGen Biotech Co., LTD, Beijing, China) according to manufacturer’s instructions. The DNA samples were qualitied by Nanodrop 2000 (Thermo Fisher Scientific, USA), and the 16S rRNA V3-V4 regions were seleceted for amplification by high fidelity polymerase chain reaction (PCR) repeated 3 times. The PCR products were further purified by AgencourtAMpure XP (Beckman Coulter, USA). The sample-specific index sequences were added into DNA library by high fidelity PCR to mixture samples, and process the bioinformatics with different label sequences to obtain raw library of each sample. These DNA labraries were quantified and validated by Qubit 3.0 (Invitrogen, Thermo Fisher Scientific, USA), and checked length of inserted fragments of the pooled samples to ensure no nonspecific amplification between 120-200 bp. Then the libraries were conducted paired-end 2 x 300 bp sequencing on an Illumina MiSeq (San Diego, CA, USA). The raw reads were filtered in Quantitative Insights Into Microbial Ecology (QIIME V 1.9.1) and compared with the Gold database. The representative operational taxonomic units (OTUs) with more than 97% similarity were conducted for further bioinformatics analysis by QIIME (V 1.9.1) and Silva database. The OTUs less than 97% similarity were considered as different species, and less than 93% - 95% similarity OTUs were considered as different genus. Rarefaction analysis was performed with vegan R package (V 2.15.3) to assess the number of OTUs in each group. Heat maps were clustered to represent the species composition of each sample and reflect the similarity and difference based on the distance by vegan R package (V 2.15.3). Kruskal–Wallis test was performed for relative abundance of different group comparisons in QIIME (V 1.9.1). The species difference analysis was performed by linear discriminant analysis (LDA) effect size (LEfSe) of beta diversity in QIIME (V 1.9.1). Principal co-ordinates (PCoA) analysis of beta diversity was performed based on weighted unifrac metrics, and the Shannon index of alpha diversity was calculated based on genus profiles in QIIME (V 1.9.1) ^[3, 4]^.

**Results**

## Mortality of each group

The eight rats in Sham+NS group all survived. The model rats were divided into BDL+NS group (n=18) and BDL+RIF group (n=16) within the post-operative first 20 days. Eight out of eighteen rats of BDL+NS group died with mortality rate of 44.4% (8/18, two rats died on the 4^th^ and 6^th^ day after operation respectively, and tow rats died every day from the post-operative 8^th^ to 10th day). Five rats died in BDL+RIF group with mortality rate of 31.3% (5/16, two rats died on the 6^th^ day after operation, and three rats died on the post-operative 8^th^, 9^th^, and 11^th^ day). The causes of death included the abdominal infection secondary to biliary fistula and gavage misoperation. On the 20^th^ day after operation, the BDL+NS group (n=10) was further equally divided into two subgroups (BDL+NS group and BDL+LAC group), and the BDL+RIF group was subdivided into BDL+RIF group (n=5) and BDL+LAC+RIF group (n=6). 2 rats of BDL+LAC+RIF group died because of the gavage misoperation on the post-operative 20^th^ day, thus four rats of BDL+LAC+RIF group were involved for micro-PET/CT imaging.

**References**

1. Fookes CJ, Pham TQ, Mattner F, Greguric I, Loc'h C, Liu X, et al. Synthesis and biological evaluation of substituted [18F]imidazo[1,2-a]pyridines and [18F]pyrazolo[1,5-a]pyrimidines for the study of the peripheral benzodiazepine receptor using positron emission tomography. J Med Chem. 2008, 51(13):3700-3712.
2. Kong X, Luo S, Wang YF, Yang GF, Lu GM, Zhang LJ. [^18^F]PBR146 and [^18^F]DPA-714 in vivo Imaging of Neuroinflammation in Chronic Hepatic Encephalopathy Rats. Front Neurosci. 2021, 15:678144.
3. Li H, Xu H, Li Y, Jiang Y, Hu Y, Liu T, et al. Alterations of gut microbiota contribute to the progression of unruptured intracranial aneurysms. Nat Commun. 2020, 11(1):3218.
4. Mahnert A, Moissl-Eichinger C, Zojer M, Bogumil D, Mizrahi I, Rattei T, et al. Man-made microbial resistances in built environments. Nat Commun. 2019, 10(1):968.

**Supplementary figures and figure legends**


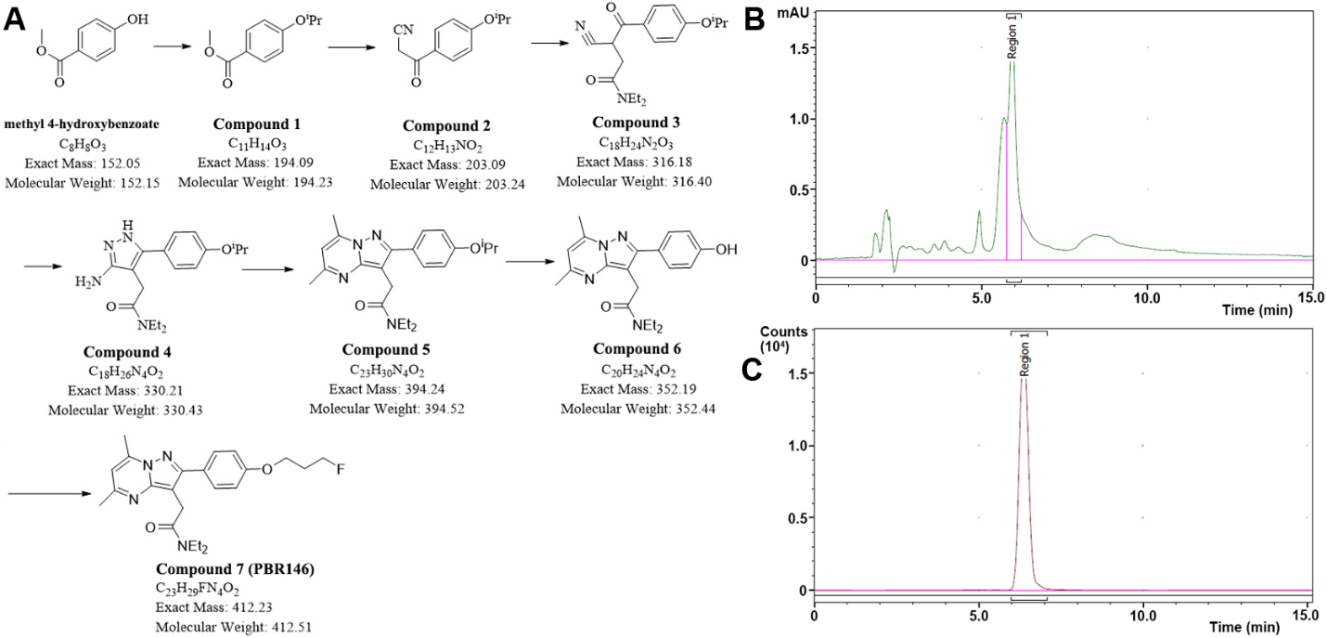


## Supplementary figure S1. The synthetic route of PBR146 compound and HPLC results of [^19^F]PBR146 and [^18^F]PBR146.

Panel **(A)** shows the synthetic route of PBR146 compound. The retention time of absorption peak of [^19^F]PBR146 and [^18^F]PBR146 was about 5.90 min **(B)** and 6.24 min **(C)** respectively, the peak magnitude was different. HPLC = high performance liquid chromatography.


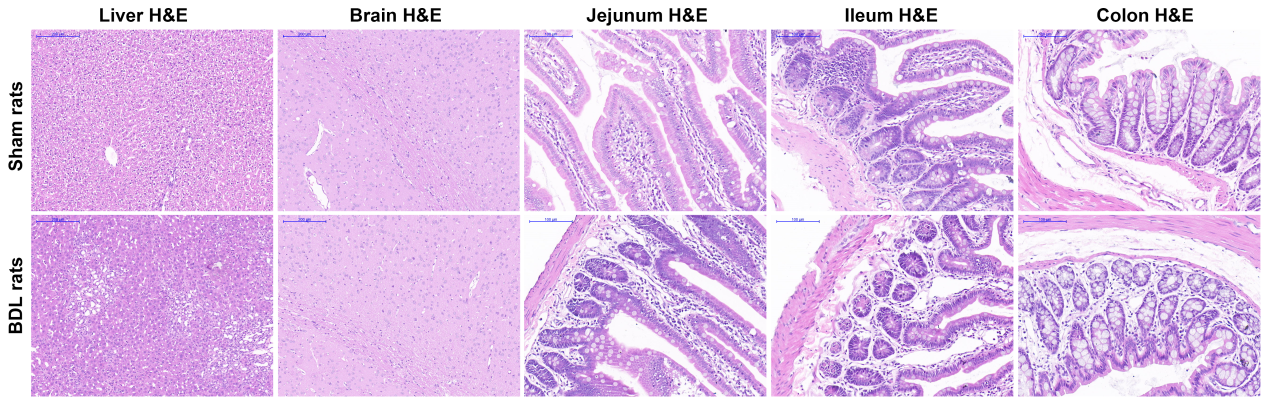


## Supplementary figure S2. The representative H&E staining findings in Sham and BDL rats.

The representative liver H&E staining (100× magnification) image shows normal hepatic histology in the sham operated rats, while the staining images show expanded bile duct leading to destroyed hepatic cords with inflammatory infiltration in the BDL rats. H&E staining (100×) of brain tissue shows no difference between Sham and BDL rats. The jejunum, ileum, and colon tissues H&E staining images (200×) show normal intestinal structures in Sham and BDL rats, which were not related with treatments to HE. H&E = hematoxylin-eosin; BDL = bile duct ligation; HE = hepatic encephalopathy.


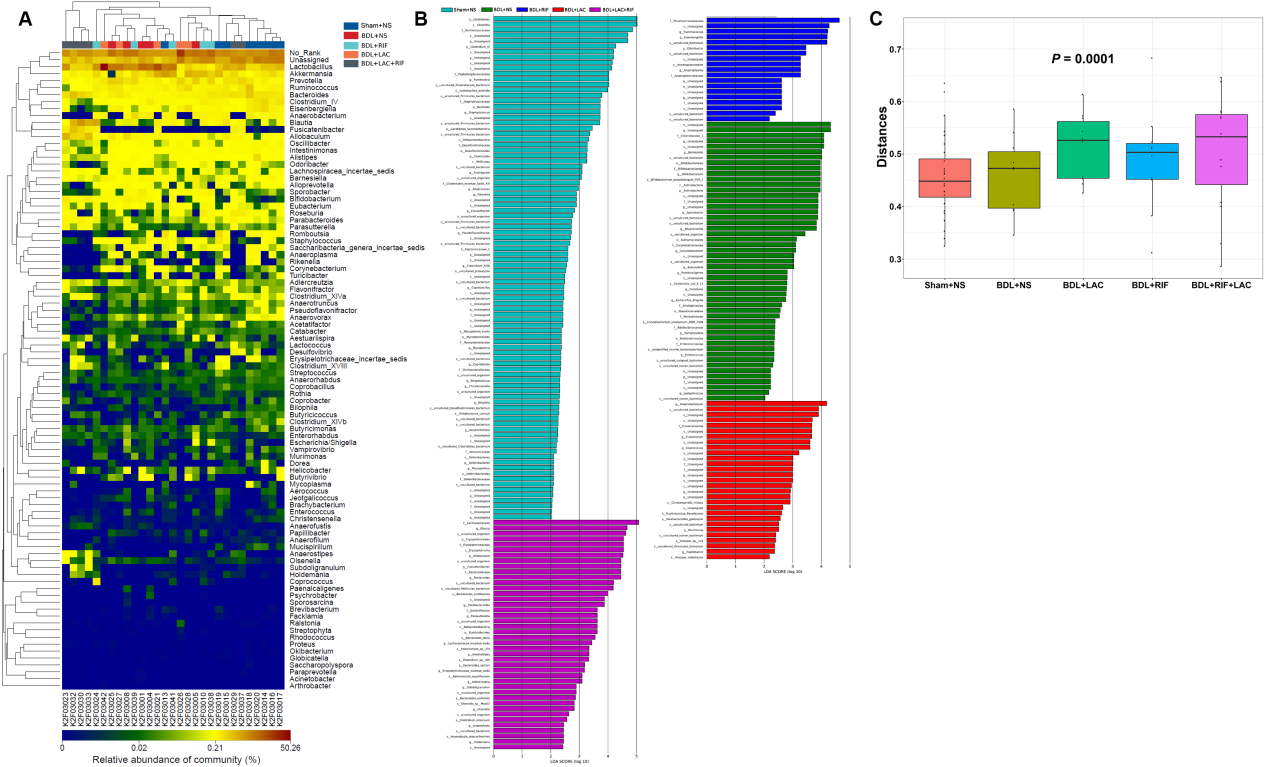


## Supplementary figure S3. The heat map, LDA pathways, and ANOSIM boxes of five groups.

Heat map of top 100 relative abundances community of each sample in five groups (**A**). The LDA pathways with significant differential abundance among five groups (**B**), LDA score higher than 2 indicated a higher relative abundance in the corresponding group than in other groups. The differences in community composition evaluated by ANOSIM based on the genus profiles in five groups (**C**). In box plot (**C**), the boxes represent the IQRs between the first and third quartiles, and the line inside the box represents the median, whiskers represent the lowest and highest values within 1.5 times IQR from the first and third quartiles, and the dots outside whiskers represent the outliers.

BDL = bile duct ligation; NS = normal saline; RIF = rifaximin; LAC = lactulose; LDA = linear discriminant analysis; ANOSIM = analysis of similarity.

**Supplementary table**

## Supplementary table S1. Comparison of [^18^F]PBR146 uptake values in brain regions and several organs among different groups (%ID/g)

| **Organs / brain regions** | **Sham+NS group (n=5)** | **BDL+NS group (n=5)** | **BDL+RIF group (n=5)** | **BDL+LAC group (n=5)** | **BDL+RIF+LAC group (n=4)** | ***P*** |
| --- | --- | --- | --- | --- | --- | --- |
| **Organs** |  |  |  |  |  |  |
| Lung | 0.469±0.044 | 0.654±0.196 | 0.496±0.182 | 0.670±0.248 | 0.570±0.091 | 0.279 |
| Myocardium | 1.420±0.130 | 1.140±0.167 | 1.100±0.071 | 1.300±0.187 | 1.220±0.269 | 0.055 |
| Liver | 0.254±0.065^bbb^ | 0.514±0.044^aaa^ | 0.496±0.073^aa^ | 0.660±0.158^aaa^ | 0.448±0.107^aa^ | <0.001*** |
| Kidney | 0.992±0.339 | 1.140±0.115 | 0.990±0.060 | 1.168±0.304 | 1.005±0.062 | 0.698 |
| **Global brain** | 0.079±0.006^bbb^ | 0.132±0.016^aaa^ | 0.082±0.008^bbb^ | 0.095±0.025^bb^ | 0.083±0.005^bbb^ | <0.001*** |
| **Brain regions** |  |  |  |  |  |  |
| Accumbens_L | 0.052±0.014^bb^ | 0.100±0.016^aa^ | 0.069±0.014^b^ | 0.071±0.028^b^ | 0.061±0.013^bb^ | 0.009** |
| Accumbens_R | 0.046±0.013^bb^ | 0.080±0.028^a^ | 0.039±0.008^bb^ | 0.048±0.015^b^ | 0.052±0.024^b^ | 0.026* |
| Amygdala_L | 0.106±0.027 | 0.150±0.031 | 0.125±0.032 | 0.109±0.037 | 0.122±0.026 | 0.225 |
| Amygdala_R | 0.086±0.019^bb^ | 0.143±0.026^aa^ | 0.121±0.033^a^ | 0.102±0.004^b^ | 0.114±0.025 | 0.016* |
| Striatum_L | 0.059±0.021^bb^ | 0.086±0.015^aa^ | 0.058±0.010^bb^ | 0.063±0.009^b^ | 0.064±0.009^b^ | 0.026* |
| Striatum_R | 0.056±0.018^bb^ | 0.086±0.022^aa^ | 0.055±0.006^bb^ | 0.068±0.014 | 0.062±0.007^b^ | 0.030* |
| Auditory Cortex_L | 0.090±0.015 | 0.103±0.036 | 0.105±0.024 | 0.095±0.021 | 0.085±0.009 | 0.655 |
| Auditory Cortex_R | 0.128±0.027 | 0.131±0.055 | 0.113±0.012 | 0.121±0.032 | 0.109±0.048 | 0.876 |
| Cingulate Cortex_L | 0.056±0.007 | 0.089±0.029 | 0.059±0.010 | 0.078±0.028 | 0.058±0.017 | 0.076 |
| Cingulate Cortex_R | 0.071±0.007 | 0.102±0.026 | 0.062±0.007 | 0.067±0.015 | 0.069±0.029 | 0.019* |
| Entorhinal Cortex_L | 0.132±0.043 | 0.163±0.028 | 0.128±0.026 | 0.136±0.042 | 0.139±0.057 | 0.665 |
| Entorhinal Cortex_R | 0.123±0.020 | 0.153±0.029 | 0.145±0.050 | 0.145±0.038 | 0.137±0.034 | 0.743 |
| Frontal Association Cortex_L | 0.075±0.030 | 0.120±0.034 | 0.066±0.028 | 0.069±0.030 | 0.060±0.045 | 0.072 |
| Frontal Association Cortex_R | 0.059±0.041 | 0.107±0.046 | 0.059±0.018 | 0.065±0.024 | 0.060±0.017 | 0.130 |
| Insular Cortex_L | 0.089±0.028 | 0.110±0.017 | 0.084±0.018 | 0.078±0.024 | 0.088±0.017 | 0.215 |
| Insular Cortex_R | 0.099±0.035 | 0.117±0.026 | 0.085±0.005 | 0.092±0.039 | 0.093±0.013 | 0.423 |
| Medial Prefrontal Cortex_L | 0.056±0.013 | 0.091±0.034 | 0.059±0.014 | 0.064±0.016 | 0.059±0.022 | 0.096 |
| Medial Prefrontal Cortex_R | 0.048±0.019^bb^ | 0.083±0.013^aa^ | 0.065±0.020 | 0.061±0.009^b^ | 0.058±0.018^b^ | 0.041* |
| Motor Cortex_L | 0.069±0.025^b^ | 0.093±0.017^a^ | 0.057±0.010^bb^ | 0.069±0.016^b^ | 0.067±0.004^b^ | 0.032* |
| Motor Cortex_R | 0.072±0.012 | 0.102±0.032 | 0.063±0.016 | 0.069±0.026 | 0.063±0.014 | 0.056 |
| Orbitofrontal Cortex_L | 0.065±0.019 | 0.104±0.019 | 0.072±0.023 | 0.083±0.038 | 0.077±0.014 | 0.165 |
| Orbitofrontal Cortex_R | 0.069±0.018 | 0.085±0.022 | 0.060±0.017 | 0.085±0.019 | 0.069±0.010 | 0.164 |
| Para Cortex_L | 0.058±0.011 | 0.075±0.012 | 0.065±0.028 | 0.057±0.025 | 0.058±0.013 | 0.522 |
| Para Cortex_R | 0.063±0.009 | 0.084±0.028 | 0.063±0.022 | 0.066±0.025 | 0.068±0.029 | 0.595 |
| Retrosplenial Cortex_L | 0.087±0.012 | 0.115±0.037 | 0.075±0.017 | 0.089±0.019 | 0.088±0.032 | 0.188 |
| Retrosplenial Cortex_R | 0.081±0.022^b^ | 0.117±0.021^a^ | 0.081±0.014^b^ | 0.107±0.021 | 0.096±0.022 | 0.042* |
| Somatosensory Cortex_L | 0.056±0.008^bb^ | 0.075±0.010^aa^ | 0.058±0.008^b^ | 0.058±0.008^b^ | 0.068±0.014 | 0.025* |
| Somatosensory Cortex_R | 0.070±0.010 | 0.093±0.028 | 0.065±0.009 | 0.074±0.009 | 0.070±0.010 | 0.078 |
| Visual Cortex_L | 0.088±0.036 | 0.112±0.025 | 0.082±0.019 | 0.100±0.048 | 0.082±0.024 | 0.567 |
| Visual Cortex_R | 0.095±0.042 | 0.123±0.022 | 0.100±0.023 | 0.110±0.036 | 0.086±0.030 | 0.476 |
| Hippocampus Antero Dorsal_L | 0.056±0.018^bb^ | 0.092±0.027^aa^ | 0.053±0.016^bb^ | 0.058±0.008^bb^ | 0.064±0.010^b^ | 0.016* |
| Hippocampus Antero Dorsal_R | 0.064±0.018^bbb^ | 0.104±0.016^aaa^ | 0.064±0.008^bbb^ | 0.073±0.009^bb^ | 0.062±0.013^bbb^ | 0.001** |
| Hippocampus Posterior_L | 0.091±0.020 | 0.118±0.041 | 0.081±0.018 | 0.099±0.028 | 0.099±0.015 | 0.320 |
| Hippocampus Posterior_R | 0.084±0.023^bb^ | 0.120±0.021^aa^ | 0.085±0.012^bb^ | 0.084±0.004^bb^ | 0.090±0.020^b^ | 0.016* |
| Hypothalamus_L | 0.111±0.025^b^ | 0.166±0.044^a^ | 0.096±0.021^bb^ | 0.116±0.047^b^ | 0.094±0.035^bb^ | 0.038* |
| Hypothalamus_R | 0.086±0.033^bb^ | 0.141±0.023^aa^ | 0.082±0.028^bb^ | 0.103±0.034 | 0.097±0.025^b^ | 0.038* |
| Olfactory_L | 0.121±0.029 | 0.162±0.035 | 0.138±0.044 | 0.119±0.007 | 0.139±0.016 | 0.201 |
| Olfactory_R | 0.092±0.037 | 0.135±0.041 | 0.095±0.010 | 0.105±0.031 | 0.103±0.041 | 0.320 |
| Colliculus Superior_L | 0.049±0.029 | 0.081±0.031 | 0.058±0.029 | 0.050±0.012 | 0.056±0.022 | 0.331 |
| Colliculus Superior_R | 0.062±0.024 | 0.104±0.058 | 0.076±0.015 | 0.061±0.026 | 0.060±0.013 | 0.213 |
| Midbrain_L | 0.051±0.016 | 0.083±0.045 | 0.052±0.018 | 0.057±0.015 | 0.065±0.013 | 0.266 |
| Midbrain_R | 0.065±0.019 | 0.091±0.035 | 0.056±0.023 | 0.055±0.020 | 0.055±0.019 | 0.135 |
| Ventral Tegmental Area_L | 0.089±0.013^b^ | 0.126±0.022^a^ | 0.081±0.029^bb^ | 0.094±0.017^b^ | 0.084±0.023^bb^ | 0.027* |
| Ventral Tegmental Area_R | 0.067±0.020 | 0.111±0.050 | 0.062±0.011 | 0.095±0.046 | 0.087±0.024 | 0.181 |
| Cerebellum-Grey_L | 0.114±0.026 | 0.154±0.012 | 0.095±0.014 | 0.110±0.059 | 0.108±0.017 | 0.081 |
| Cerebellum-Grey_R | 0.108±0.039 | 0.140±0.017 | 0.103±0.018 | 0.126±0.048 | 0.107±0.019 | 0.338 |
| Cerebellum-White_L | 0.121±0.028 | 0.175±0.056 | 0.130±0.038 | 0.136±0.023 | 0.129±0.021 | 0.192 |
| Cerebellum-White_R | 0.119±0.032^b^ | 0.165±0.038^a^ | 0.117±0.022 | 0.129±0.026 | 0.109±0.013 | 0.048* |
| Colliculus Inferior_L | 0.052±0.025 | 0.093±0.051 | 0.049±0.036 | 0.068±0.019 | 0.053±0.020 | 0.237 |
| Colliculus Inferior_R | 0.078±0.025 | 0.109±0.055 | 0.074±0.025 | 0.088±0.035 | 0.078±0.026 | 0.538 |
| Thalamus_L | 0.047±0.009 | 0.066±0.019 | 0.049±0.007 | 0.064±0.023 | 0.056±0.010 | 0.251 |
| Thalamus_R | 0.051±0.012 | 0.072±0.035 | 0.053±0.010 | 0.064±0.007 | 0.054±0.015 | 0.372 |
| Pituitary | 0.342±0.088 | 0.463±0.176 | 0.316±0.074 | 0.407±0.101 | 0.399±0.068 | 0.283 |
| Cerebellum-blood | 0.177±0.052 | 0.242±0.055 | 0.209±0.037 | 0.213±0.069 | 0.219±0.025 | 0.413 |
| Central Canal-Periaqueductal Gray | 0.049±0.015 | 0.063±0.040 | 0.055±0.020 | 0.056±0.019 | 0.049±0.009 | 0.878 |
| Pons | 0.097±0.033 | 0.144±0.063 | 0.090±0.019 | 0.125±0.039 | 0.129±0.020 | 0.200 |
| Septum | 0.075±0.028 | 0.087±0.031 | 0.085±0.022 | 0.077±0.023 | 0.067±0.030 | 0.816 |
| Medulla | 0.128±0.024 | 0.156±0.042 | 0.112±0.022 | 0.115±0.012 | 0.124±0.022 | 0.125 |

Note: Values significantly different from Sham+NS group are indicated by ‘a’ and from BDL+NS group by ‘b’. * *P*<0.05, ***P*<0.01, and ****P*<0.001; ^a^*P*<0.05, ^aa^*P*<0.01, and ^aaa^*P*<0.001; ^b^*P*<0.05, ^bb^*P*<0.01, and ^bbb^*P*<0.001 were regarded as statistically significant.

BDL = bile duct ligation; NS = normal saline; RIF = Rifaximin; LAC = Lactulose; L = left; R = right.
